# Premature lethality, hyperactivity, and aberrant phosphorylation in transgenic mice expressing a constitutively active form of Fyn

**DOI:** 10.3389/fnmol.2014.00040

**Published:** 2014-05-13

**Authors:** Di Xia, Jürgen Götz

**Affiliations:** Clem Jones Centre for Ageing Dementia Research, Queensland Brain Institute, The University of QueenslandBrisbane, QLD, Australia

**Keywords:** Alzheimer, dendrite, Fyn kinase, palmitoylation, phosphorylation, spine, tau

## Abstract

The kinase Fyn, the microtubule-associated protein tau and the peptide amyloid-β (Aβ) constitute a toxic triad in Alzheimer's disease (AD). Tau's subcellular localization is mainly regulated by phosphorylation whereas Fyn's localization is dictated by palmitoylation targeting it to the plasma membrane in a reversible manner. We have previously shown that tau is required for Fyn to be targeted to the dendritic spine. We had also shown that a truncated form of tau (Δtau) that accumulates in the cell soma is capable of trapping Fyn and preventing it from entering the spine. Here we determined that palmitoylation is required for Fyn's membrane and spine localization. We further evaluated the functional consequences of neuronal over-expression of the constitutively active Y531F mutant form of Fyn (FynCA) in transgenic mice. We found that the FynCA transgenic mice displayed a reduced weight, a massively reduced lifespan and a high level of hyperactivity. The lifespan of the FynCA mice was only slightly extended by crossing them with Δtau transgenic mice, possibly reflecting differences in expression patterns of the transgenes and high levels of transgenic FynCA compared to endogenous Fyn. Analysis of synaptosomes revealed that FynCA accumulated at high levels in the spine, resulting in increased levels of the NMDA receptor subunit NR2b phosphorylated at residue Y1472. Tau was strongly phosphorylated at the AT8 epitope S202/T205 as shown by Western blot and immunohistochemistry indicating that an increased tyrosine kinase activity of Fyn has down-stream consequences for serine/threonine-directed phosphorylation.

## Introduction

**Figure 1:**
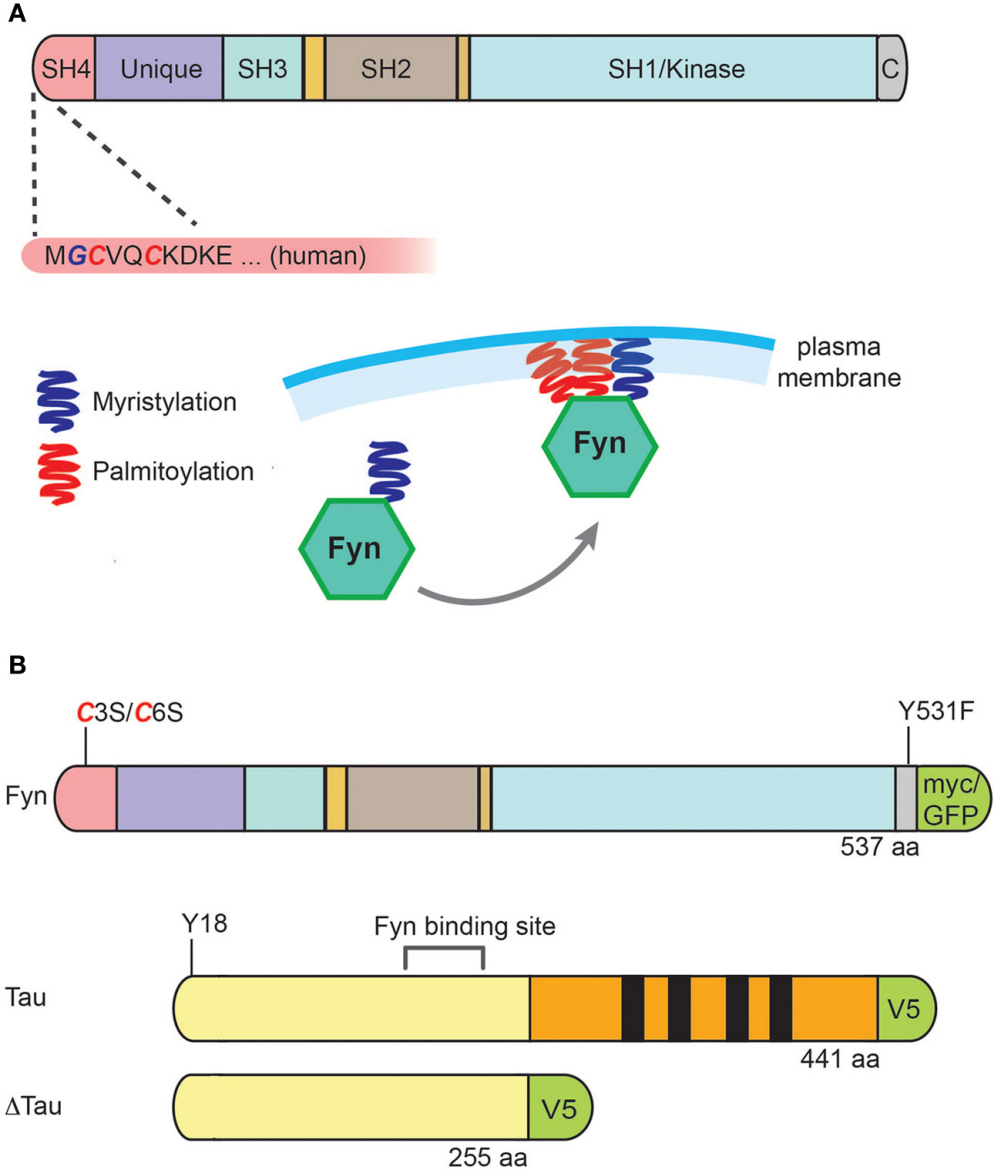
**Structure of Fyn, its membrane localization, and constructs used for expression of Fyn, tau and ΔTau**. **(A)** Fyn is a 59 kDa protein that contains an amino-terminal Src-homology (SH) region with acylation sites, a unique domain, an SH3 domain (with which it interacts with PXXP motifs), an SH2 domain (with which it interacts with phospho-tyrosine), an SH1/kinase domain, and a carboxy-terminal regulatory tail. The most amino-terminal glycine residue can be myristoylated, which occurs co-translationally on free ribosomes. In addition, Fyn's amino-terminal cysteines C3 and C6 can be palmitoylated, and it is this acylation reaction that anchors the kinase to the plasma membrane. **(B)** Expression constructs for human Fyn were either wild-type, C3S/C6S to prevent palmitoylation, or Y531F to generate a constitutively active form of Fyn (FynCA). Tau was expressed either as full-length [with the longest human tau isoform, hTau40, containing four microtubule-binding domains (MBDs) indicated in black] or a truncated form (Δtau) lacking the MBDs. For detection of tau and Fyn, myc, GFP and V5 were used as tags as indicated.

In Alzheimer's disease (AD), serine/threonine-directed phosphorylation has attracted significantly more attention than tyrosine-directed phosphorylation (Götz et al., 2013). One of the reasons is that the microtubule-associated protein tau, a protein implicated in AD, contains 80 serines and threonines, many of which have been shown to be phosphorylated under disease conditions, compared to only 5 tyrosine residues (Chen et al., 2004). When tau transgenic mice as a model of AD are analyzed this routinely includes the assessment of the phosphorylation status of relevant serine and threonine residues, but rarely that of tyrosines. Similarly, to establish the sequence of tau-related cytoskeletal changes in AD, the serine/threonine-directed antibody AT8 and not a tyrosine-directed antibody has been used (Braak et al., 1994). However, it is increasingly appreciated that tyrosine phosphorylation has also an important role in AD (Boehm, 2013). Of tau's five tyrosine residues, Y18 is particularly interesting because it is specifically phosphorylated by the tyrosine-directed kinase Fyn, a member of the Src family of non-receptor tyrosine kinases (http://cnr.iop.kcl.ac.uk/hangerlab/tautable) (Figure 1A).

Under physiological conditions, the majority of tau is localized to the axon, whereas tau's “counterpart,” MAP2, is mainly localized to the dendrite. However, as we have found recently, tau is also localized, albeit at lower levels, to the dendrite where it serves an important function that cannot be taken over by MAP2 (Ittner et al., 2010): Tau is required to target Fyn to the dendritic spine where it phosphorylates the NMDA receptor, a prerequisite for recruiting the postsynaptic density (PSD) (95 kD) protein (PSD-95) into a protein complex. This complex then mediates the excitotoxic signaling triggered by Aβ, a peptide that aggregates and forms amyloid plaques in AD brains. In the absence of tau or when a form of tau is expressed that accumulates in the cell soma and is excluded from the dendrite (Δtau, Figure 1B), Fyn is prevented from entering the spine and thereby, Aβ cannot excitotoxically signal through the NMDA receptor (either directly, or indirectly) any more (Ittner et al., 2010). When a full-length form of tau is expressed that carries the pathogenic P301L mutation found in frontotemporal dementia, excitotoxic signaling is augmented because of an increased dendritic localization of phosphorylated mutant tau and consequently, increased levels of dendritic Fyn (Ittner and Götz, 2011). These data are supported by the finding that Aβ causes missorting of endogenous tau into the dendritic compartment (Zempel et al., 2010). Not surprisingly, overexpression of wild-type murine Fyn induces synaptic and cognitive impairments in a transgenic mouse model with Aβ accumulation (Chin et al., 2005), and deletion of Fyn ameliorates some of the phenotypes induced by Aβ (Chin et al., 2004). Together, this established the notion of a toxic triad of the three molecules, tau, Fyn and Aβ (Haass and Mandelkow, 2010), presenting Aβ as the trigger, and tau as the bullet in AD pathogenesis (Bloom, 2014).

Fyn is a 59 kDa protein that exists as two isoforms due to alternative splicing of exon 7, with FynT being expressed in T-cells, and FynB in the brain and additional organs (Kramer-Albers and White, 2011). Fyn contains, in this order, an amino-terminal Src-homology (SH) region with acylation sites, a unique domain, an SH3 domain (with which it interacts with PXXP motifs), an SH2 domain (with which it interacts with phospho-tyrosine), an SH1/kinase domain, and a carboxy-terminal regulatory tail (Figure 1A). The most amino-terminal glycine residue can be myristoylated, which occurs co-translationally on free ribosomes. In addition, Fyn's amino-terminal cysteines, C3 and C6, can be palmitoylated, and it is this acylation reaction that anchors the kinase to the plasma membrane. Palmitoylation in general enhances the hydrophobicity of proteins and contributes to their membrane association. In contrast to myristoylation, palmitoylation is usually reversible (because the bond between palmitic acid and protein is often a thioester bond) (Figure 1A). A good example of another palmitoylated protein in the dendritic spine is PSD-95. Interestingly, palmitoylation of Fyn seems to direct the SH4 domain away from the membrane, which may be important for the interaction of Fyn with target proteins (Rawat and Nagaraj, 2010).

The activity of Fyn is regulated by differential phosphorylation at multiple sites (Kramer-Albers and White, 2011). In its inactive state, the regulatory tyrosine at the carboxy-terminus (Y531 in mouse FynB) is phosphorylated, forming an intra-molecular bond with the SH2 domain of the kinase. This phosphorylation negatively regulates kinase activity (Nada et al., 1993). Furthermore, the SH3 domain binds intra-molecularly to a linker region located between the SH2 and the kinase domain. This keeps the kinase in an inactive closed conformation in which both protein binding domains are occupied. Upon dephosphorylation of the carboxy-terminal regulatory tyrosine residue by tyrosine phosphatases, the intramolecular binding to the SH2 domain is abolished. Loss of SH2-dependent folding alters the conformation of the kinase to generate a more open form making the SH2 and SH3 domains available for downstream protein interactions. Furthermore, the open conformation enables an intermolecular auto-phosphorylation of Y420 that stabilizes the active state of the catalytic site (Roskoski, 2004). Src kinases can also be stimulated by interfering with the inhibitory intra-molecular binding of SH2 and SH3 domains which both can bind to external ligands leading to an activation of the kinase (Ostareck-Lederer et al., 2002; Sette et al., 2002).

Tau interacts with Fyn at least in two ways. Firstly, Y18 is phosphorylated by Fyn, and the phosphorylated motif interacts with Fyn via Fyn's SH2 domain (Usardi et al., 2011). Secondly, Tau contains seven PXXP motifs located in the amino-terminus (all of which are retained in the Δtau construct). Of these, the seventh proline-rich RTPPKSP motif has been shown to be crucial for the interaction with the SH3 domain of Fyn and other Src non-receptor tyrosine kinases (Lee et al., 1998; Bhaskar et al., 2010; Ittner et al., 2010). Interestingly, this motif is also critical in the interaction of tau with the heterotrimeric phosphatase PP2A (Sontag et al., 2012). The PP2A regulatory subunit Bα binds to and dephosphorylates tau, and thereby regulates microtubule stability (Kins et al., 2001; Sontag et al., 2012). When Fyn is bound to the (seventh) proline-rich RTPPKSP motif that is conserved in both tau and MAP2, this inhibits the interaction of PP2A/Bα with either tau or MAP2. The corresponding synthetic RTPPKSP peptide, but not the phosphorylated RpTPPKSP version, competes with tau and MAP2 for binding to PP2A/Bα. This finding is remarkable because the down-regulation of PP2A/Bα and the deregulation of Fyn/tau interactions have been linked to enhanced tau phosphorylation in AD (Sontag et al., 2012). Why in tau knock-out mice, MAP2 that is mainly a dendritic protein cannot compensate for the absence of tau and target Fyn to the dendritic spine is not understood, especially as both MAP2 and tau can efficiently bind Fyn (Sontag et al., 2012; Lashuel et al., 2013). Not only tau but also MAP2 have been shown to bind to Fyn via Fyn's SH3 domain (Zamora-Leon et al., 2001).

Here, we aimed to address how Fyn is localized subcellularly *in vitro* and what the functional consequences are of the transgenic overexpression of a constitutively active form of Fyn, Y531F.

## Materials and methods

### Mutagenesis

The C3S/C6S double mutation was introduced by PCR into the lentiviral vector Fyn-myc pLUV6 that contains the human Fyn isoform 1 cDNA fused to a myc tag (Ittner et al., 2010). The resultant mutant construct was subcloned into the pGEM-T easy vector by TA ligation (Promega), followed by ApaI/SalI digestion and ligation into the pEGFP-N1 vector (Clontech) to generate a C3S/C6S Fyn-EGFP construct. Similarly, the Y531F mutation was cloned by PCR, with or without a myc-tag, and subcloned via a unique XhoI site into the murine Thy1.2 expression vector pEX12 for subsequent neuronal expression in transgenic mice (Ittner and Götz, 2007). The plasmids were sequenced using the service of the AEGRC sequencing facility (University of Queensland). The resulting transgenic mice were labeled FynCA.

### Cell culture

HEK293T cells were cultured in DMEM medium, supplemented with 2 mM Glutamax (Life Technologies) and 10% FBS (Sigma). Cells were seeded as 50% confluency in 6 cm culture dishes and allowed to grow for 24h before transfection. For high efficiency transfection, lipofectamine LTX (Invitrogen) was used in a 2:1 ratio to DNA. Hippocampal neurons from E18 wild-type or Tau knock-out (Tucker et al., 2001) mouse pups were plated onto poly-D-lysine coated coverslips in a 12-well plate at a density of 5000 cells/well. As a plating medium, Neurobasal medium was used, supplemented with 5% FBS (Hyclone), 2% B27 (Life Technologies), 2 mM Glutamax, and 50 U/mL penicillin/streptomycin. Neurons were switched to serum-free Neurobasal medium 24h post-seeding and half the medium was changed twice a week. Neurons were transfected at DIV 18 using lipofectamine 2000 (Invitrogen).

### Generation of transgenic mice

FynCA transgenic mice were generated by pronuclear microinjection as described previously (Ittner and Götz, 2007). Δtau74 mice have been generated previously, by removing amino acids 256–441 from the longest human tau isoform, htau40, and expressing this truncated form of tau under control of the murine Thy1.2 promoter (Ittner et al., 2010). As a tau knock-out strain, mice were used that have a GFP cassette inserted in frame into the first coding exon, resulting in a fusion protein that contains the first 31 amino acids of tau (Tucker et al., 2001). Animal experimentation has been approved by the Animal Experimentation Committee of the University of Queensland (QBI/327/11/NHMRC/ARC/BREED, QBI/027/12/ NHMRC).

### Immunohistochemistry

Mice were immersion fixed in 4% PFA rather than perfused because of their small size, brains embedded in paraffin and 7 μm sections obtained as described (Deters et al., 2008). For antigen retrieval, the sections were microwaved in AR buffer (Dako Envision Flex Target Retrieval Solution low pH #K8005) for 15 min on low power before the buffer started to boil. The sections were then left to cool for 1h in the AR buffer at RT. Blocking was done in 20% FBS, 1% BSA in TBST for 1h at RT. Primary antibodies were used over night at 4°C and secondary antibodies for 1.5h at RT. Primary antibodies were Myc-Tag rabbit mAb (Cell Signaling Technologies, #71D10, used at 1:100) and anti-Human PHF-Tau Monoclonal Antibody AT8 (Thermo Fisher, MN1020, used at 1:400). Secondary antibodies were polyclonal goat anti-rabbit IgG biotinylated (Dako, #E0432, used at 1:500) and polyclonal rabbit anti-mouse IgG biotinylated (Dako, #E0413, used at 1:500). The VectaStain Elite ABC Kit #PK6102 was used and Envision Flex DAB chromogen (Dako, #DM827) and Envision Flex Substrate Buffer (Dako, # DM823) for DAB development. Haematoxylin was used for counter-staining.

### Synaptosomal extraction and western blot analysis

Synaptosomes from the mouse forebrain (*n* = 4 for the three genotypes) were prepared using a modified volume-adjusted protocol based on a protocol described previously (Ittner et al., 2010). In brief, the tissue was homogenized in sucrose buffer (1 ml/60 mg tissue. 0.32M sucrose, 1 mM NaHCO_3_, 1 mM MgCl_2_, 0.5 mM CaCl_2_) using a tissue homogenizer. Brain homogenate was centrifuged at 1400g for 10 min and cleared by centrifugation at 720g for 10 min. 200 μL of supernatant was collected as whole brain lysate and the pellet containing cell debris and nuclei was discarded. The remaining supernatants were then centrifuged at 13,800g for 10 min to obtain the crude synaptosomes in the pellet (P). The pellet was resuspended in 200 μl Sucrose Buffer, layered over 1.8 mL pre-cooled 5% Ficoll and centrifuged at 45,000g for 45 min. The supernatant was then removed carefully and the pellet resuspended in 200 μL pre-cooled 5% Ficoll, which was then layered over 1.8 mL pre-cooled 13% Ficoll and centrifuged at 45,000g for 45 min. A milky interface containing the synaptosomes was recovered, topped-up with 5% Ficoll to 2 mL and centrifuged at 45,000g for 25 min to pellet the synaptosomes. Purified synaptosomes were further extracted sequentially with buffers of increasing stringency: pH6 (40 mM Tris-HCl, 2% Triton X-100, 0.5 mM CaCl_2_); pH8 (20 mM Tris-HCl, 1% Triton X-100); and SDS (5% SDS). The SDS buffer extract contains strongly PSD-associated proteins. This fraction was named “PSD.” All centrifugations were carried out at 4°C. For Western blot analysis, the following primary antibodies were used: total Tau (Dako, polyclonal, 1:5,000), Fyn (Fyn15; monoclonal, Santa Cruz, 1:500), PSD-95 (Millipore, 1:2,000), GAPDH (polyclonal, Millipore, 1:5,000), NR2b (polyclonal, Millipore, 1:1,000), pTyr^1472^- NR2b (polyclonal, Sigma, 1:1,000), Myc-tag (71D10, rabbit monoclonal, 1:2,000), AT8 (phospho-tau, pSer202 + pThr205, monoclonal, Thermo, 1:1,000), 12E8 (phospho-tau pSer262 (+ pS356)), polyclonal, Thermo, 1:4,000), HT7 (human Tau, monoclonal, Thermo, 1:2,000), and Actin (monoclonal, Millipore, clone C4, monoclonal, 1:5,000).

## Results

### Palmitoylation of Fyn is required for spine localization

**Figure 2:**
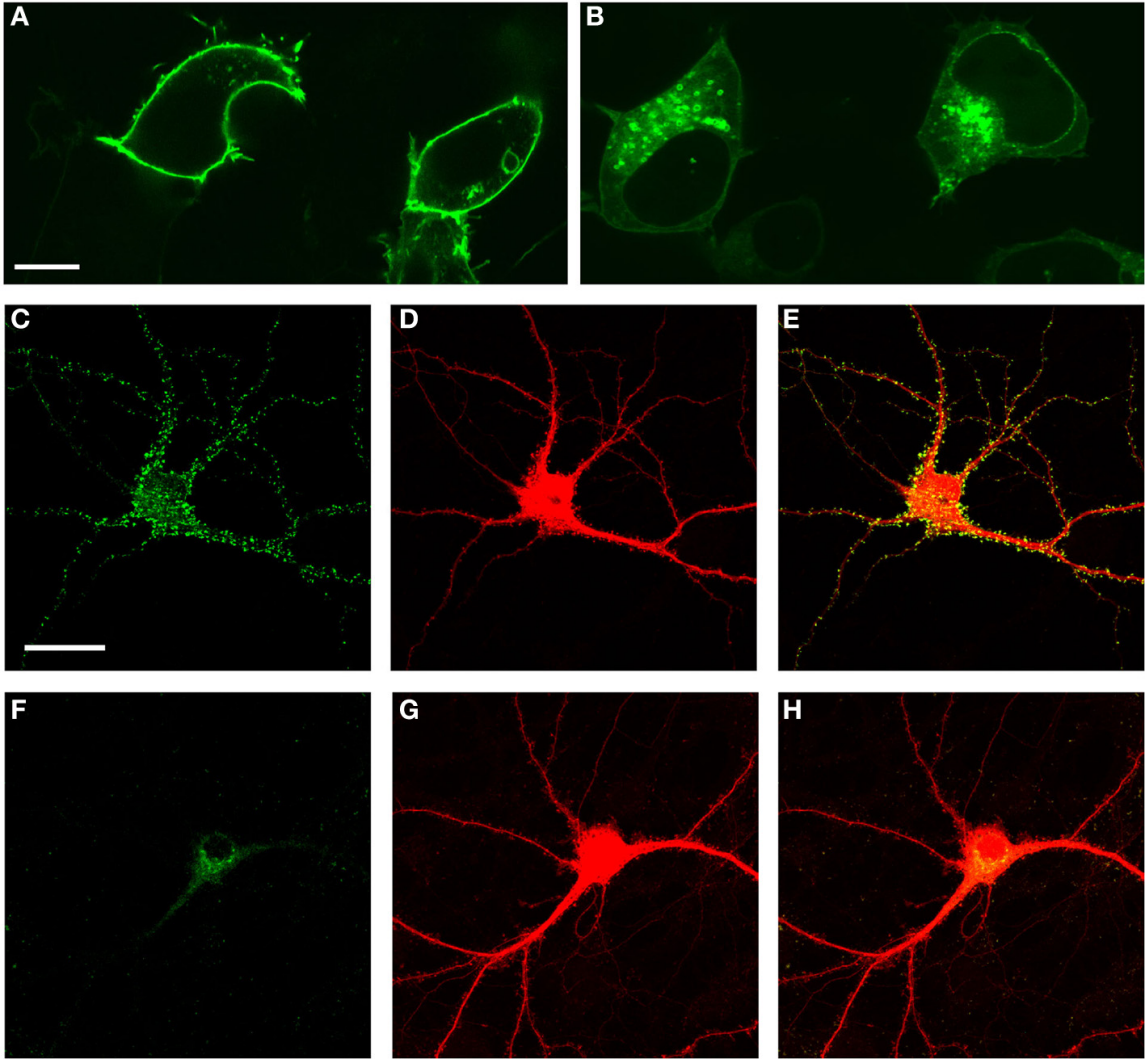
**Palmitoylation and hence, membrane association, is required for the localization of Fyn into dendritic spines**. Transfection of Fyn expression constructs in HEK293 cells **(A,B)** and primary hippocampal cultures **(C–H)** reveals that wild-type Fyn associates with the plasma membrane in HEK293 cells **(A)**, whereas mutating Fyn's two cysteines (C3/C6) that are acetylated in a palmitoylation reaction to cysteines causes Fyn's accumulation intracellularly in endosomes **(B)**. In the neuronal cultures, wild-type Fyn is localized to spines **(C–E)** whereas Fyn(C3S/C6S)-GFP is excluded from entering the spines **(F–H)**. RFP **(D,G)** was used to visualize all neuronal structures including the spines. Scale bar: 10 μm **(A,B)**; 20 μm **(C–H)**.

Fyn contains two cysteines (C3 and C6) that are acetylated in a palmitoylation reaction. To determine the role of these residues in subcellular localization we mutated the palmitoylation signal by replacing the two cysteines by serines as described (Wolven et al., 1997), and equipped Fyn with a GFP tag. When transfected into HEK293 cells, this revealed a membrane association for wild-type Fyn (Figure 2A). Fyn(C3S/C6S), in contrast, was excluded from the plasma membrane and accumulated intracellularly in endosomes (Sandilands et al., 2007) (Figure 2B). We next determined the subcellular localization in primary hippocampal cultures obtained from wild-type mouse embryos. After 21 days (DIV2) in culture, the neurons displayed a myriad of spines. We co-transfected the cultures with RFP and either Fyn-GFP or Fyn(C3S/C6S)-GFP and found that wild-type Fyn was localized to spines (Figures 2C–E) whereas Fyn(C3S/C6S)-GFP was excluded from entering the spines (Figures 2F–H). Together this reveals that palmitoylation and hence, membrane association, is required for the localization of Fyn into dendritic spines.

**Figure 3:**
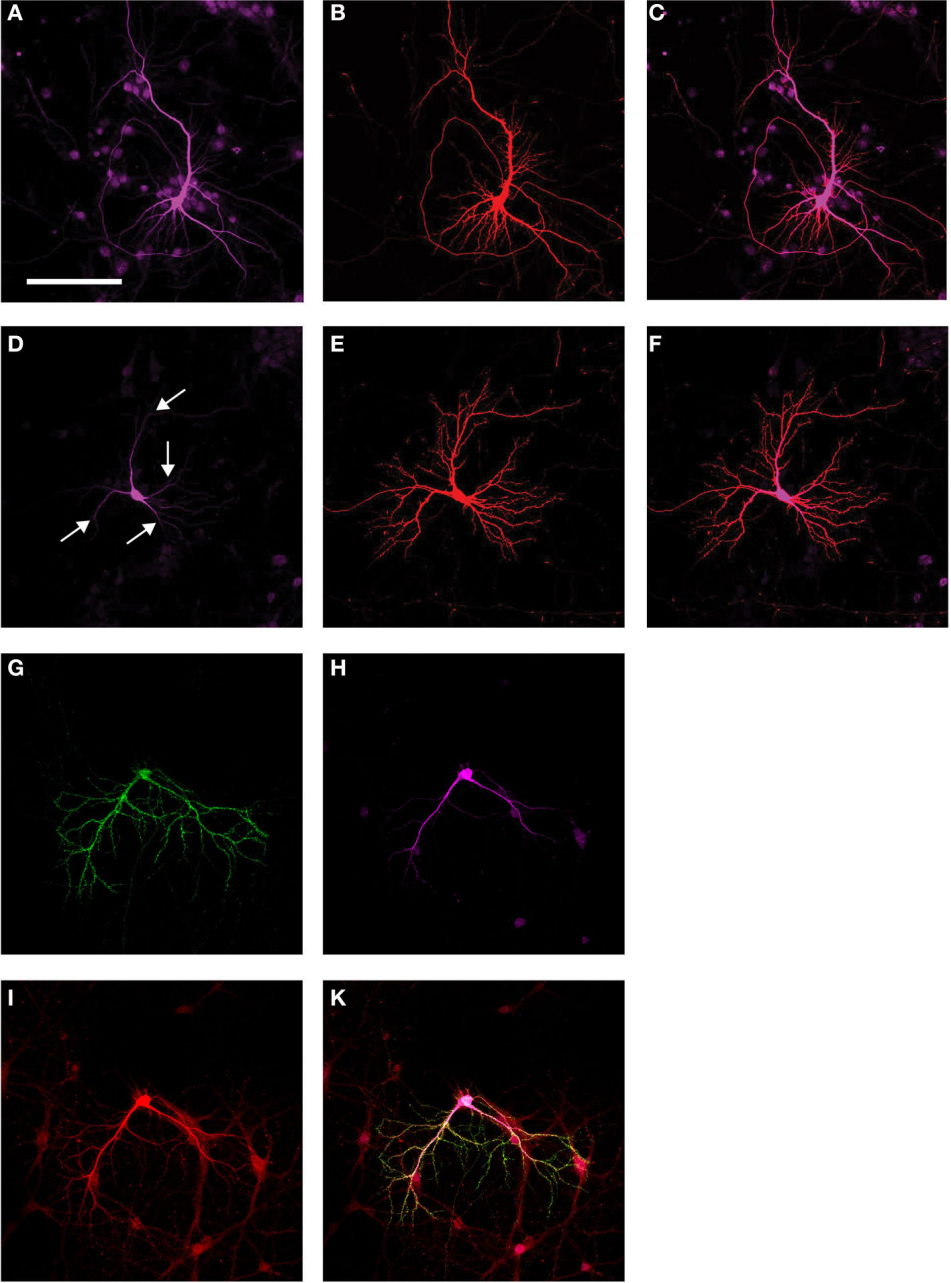
**Subcellular localization of Fyn and Δtau**. Transfection of tau knock-out primary neuronal cultures with a full-length tau construct equipped with a V5 tag (Tau-V5) **(A)** together with RFP **(B)** shows distribution of full-length tau throughout the neuron, but spines are excluded. (Merged image: **C**). Transfection of Δtau-V5 **(D)** together with RFP **(E)** reveals that Δtau is dramatically reduced from the dendritic compartment, and that levels are below detection past the secondary branch-point (as indicated by the arrows) (Merged image: **F**). When Fyn-EGFP **(G)** was over-expressed together with Δtau-V5 **(H)** and RFP **(I)** in wild-type primary neurons, different from Δtau74 mice in which transgenic over-expression of Δtau prevents endogenous Fyn from entering the spines, transfecting Δtau into primary neurons does not prevent transfected Fyn from entering the spine, possibly reflecting differences in expression levels (Merged image: **K**). Scale bar: 100 μm **(A–K)**.

We have previously shown that Δtau in Δtau74 transgenic mice is localized to the cell body, but excluded from the dendritic compartment (Ittner et al., 2010). This finding was also supported biochemically, by analyzing synaptosomal preparations and comparing Δtau localization with that of full-length tau. To determine whether Δtau is also excluded from the dendritic compartment and in particular, spines, in primary neuronal cultures, we transfected both full-length tau equipped with a V5 tag (Tau-V5) together with RFP, and Δtau-V5 together with RFP, respectively, using primary neuronal cultures derived from tau knock-out mice. This showed that full-length tau is distributed throughout the neuron, but excluded from the spines (Figures 3A–C). In contrast, Δtau was dramatically reduced from the dendritic compartment and levels were found to be below detection levels past the secondary branch-point (as indicated by the arrows) (Figures 3D–F). We next determined whether expression of Δtau would affect Fyn localization, by co-transfecting wild-type neurons with Fyn-EGFP, RFP, and Δtau-V5 (Figures 3G–K). We found that different from Δtau74 mice in which transgenic over-expression of Δtau (or knocking-out endogenous tau) prevented endogenous Fyn from entering the spines (Ittner et al., 2010), transfecting Δtau into primary neurons was not capable of preventing transfected Fyn (Fyn-EGFP) from entering the spine, possibly reflecting differences in expression levels.

### Mice with a constitutive expression of Fyn are characterized by reduced weight, hyperactivity, and premature death

**Figure 4:**
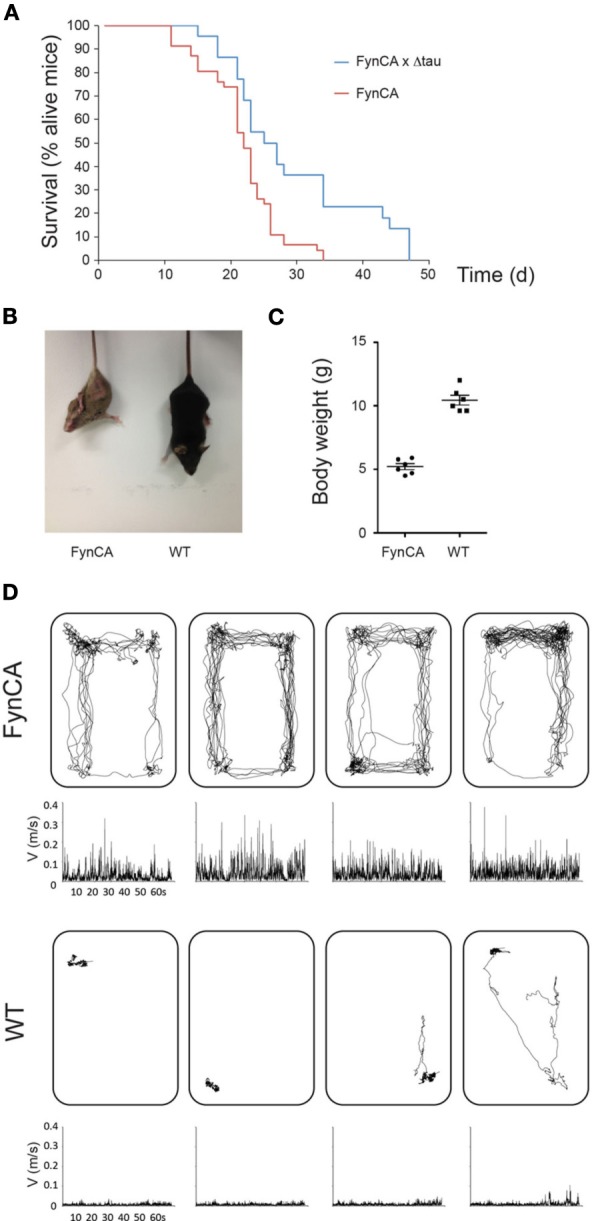
**FynCA mice are characterized by early mortality, reduced weight, and hyperactivity**. Transgene-positive offspring derived from FynCA founders #14, 35, and 55 is characterized by early lethality **(A)**. Crossing FynCA mice with Δtau mice results in a slight extension of the lifespan of the FynCA mice. The transgene-positive offspring derived from these lines tends to be smaller as shown for #55 offspring **(B)**, and to show less weight gain than the non-transgenic littermates such that by 2–3 weeks of age they weigh only half of their non-transgenic littermates (Student's *t*-test, *P* < 0.0001) **(C)**. Tracking of four FynCA transgenic mice (top) and four non-transgenic littermates (bottom) at 18 days of age. The FynCA mice run in circles and keep this hyperactivity up for hours unless being put back to their litter **(D)**.

In brain, Fyn is expressed in both neurons and glial cells, and in both cell-types, tau has an important function in regulating Fyn (Klein et al., 2002; Ittner et al., 2010). To establish a mouse model with constitutive expression of Fyn in neurons, we introduced the Y531F mutation into the human Fyn cDNA and cloned this “FynCA” cDNA into the mThy1.2 expression vector for neuronal expression, generating constructs that either contained or did not contain the myc tag for subsequent detection (Figure 1A). We generated a series of transgenic founders by pronuclear injection of C57BL/6 × DBA/2 oocytes: four (#11, 15, 16, and 17) without a myc tag, and 10 (#4, 12, 13, 14, 20, 22, 24, 35, 55, and 58) with a tag. We screened the offspring obtained from these FynCA founder animals by Western blotting and found that whenever the transgene-positive offspring died early (what happened around 3 weeks of age), the transgene was expressed at detectable levels, whereas when the mice did not die at an early age the offspring of that particular founder did not express the transgene at detectable levels (Figure 4A). We specifically identified founders #14, 35, and 55 as generating offspring characterized by early lethality. The transgene-positive offspring derived from these lines was significantly smaller, as shown for #55 offspring (Figure 4B), and showed less weight gain than the non-transgenic littermates such that by 2–3 weeks of age they would only have half the weight of non-transgenic littermates (Figure 4C). The transgenic mice displayed persistent tremor and lack of coordination while moving, as well as pronounced hyperactivity and an altered locomotion behavior (Supplementary movie, shown at 18 days of age). More specifically, when separated from the litter they would start running in circles in the cage for hours, and this could only be halted when the transgenic mice were put back to the litter. Interestingly, only a slight separation from the litter was sufficient to re-instigate this type of behavior, suggesting high levels of anxiety in FynCA mice. To complement the movie, we recorded the path of four mice each aged 18 days of age for 1 min, after the mice had been kept isolated in a box for 1h (Figure 4D).

### Partial rescue of FynCa mice by crossing onto a Δtau background

**Figure 5:**
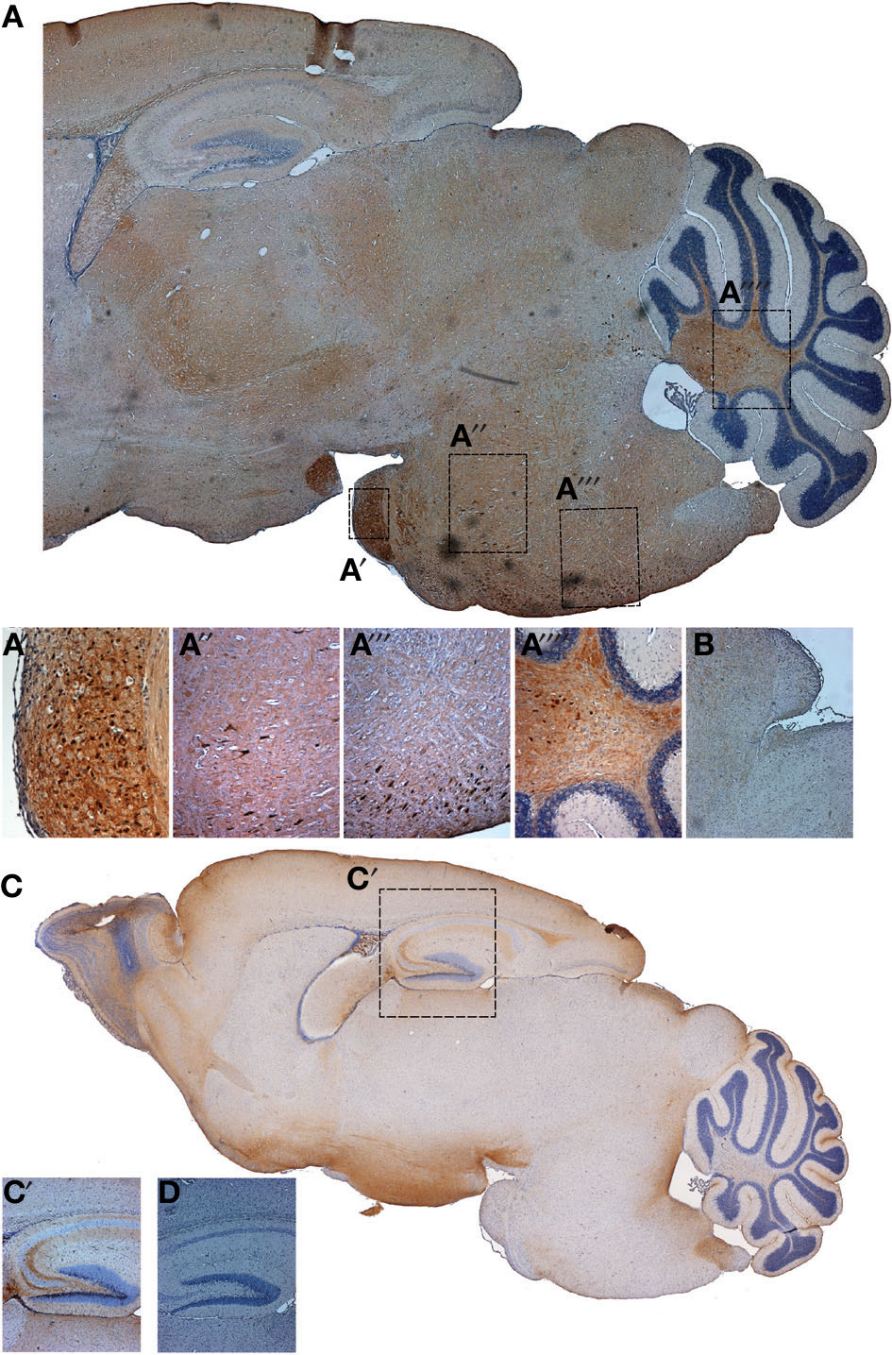
**Fyn expression in FynCA mice and tau phosphorylation**. A myc-specific antibody was used to reveal Fyn expression in FynCA-transgenic mice **(A)**, using non-transgenic littermates as controls **(B)**. Phosphorylation of endogenous tau was revealed using the Ser202/Thr205-specific antibody AT8, analyzing FynCA-transgenic mice **(C)** and non-transgenic littermate controls **(D)**.

**Figure 6:**
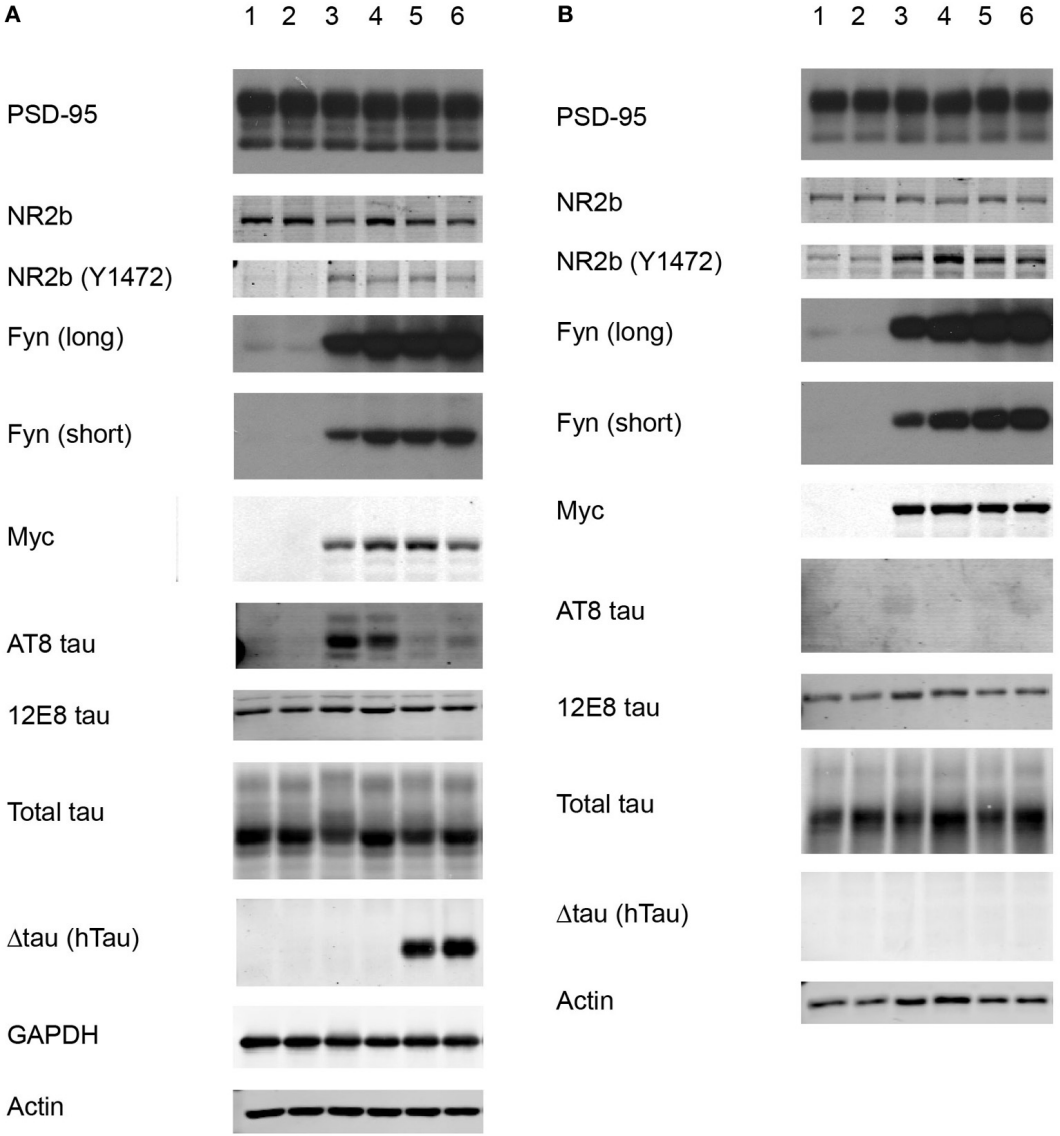
**Western blot of total brain extract and synaptosomal fractions**. We compared wild-type mice (1,2), FynCA mice (3,4), and FynCA mice crossed onto a Δtau background (5,6). **(A)** Total extracts and synaptosomal PSD (SDS) fractions **(B)** were analyzed with antibodies for GAPDH, actin, Fyn (short and long exposure), Myc, PSD-95, NR2b, NR2b phosphorylated at Y1472, total tau, human tau (HT7) detecting Δtau, and phospho-tau (AT8, 12E8).

We next analyzed the offspring of the FynCA founder animals both by immuno-histochemistry (Figure 5) and Western blotting (Figure 6) using a monoclonal anti-Fyn antibody. The latter revealed a strong expression of Fyn (59 kDa) in mouse brains derived from the FynCA founders #14, 35, and 55. As shown for offspring of founder #55, levels of Fyn in FynCA transgenic mice were approximately six-fold higher than those for endogenous murine Fyn in wild-type mice (Figure 6A). The affinity of the antibody for human and murine Fyn is expected not to differ because of a 100% homology of the epitope used for immunization as well as flanking sequences. We next determined the expression pattern of FynCA in #55 offspring by immunohistochemistry using a rabbit anti-myc antibody to visualize FynCA expression. We found that FynCA was widely expressed in FynCA transgenic brain (Figure 5A), using non-transgenic littermates as controls (Figure 5B). Prominent expression of the FynCA transgene was found in neurites and fiber tracts as shown for the hippocampus and the fimbria fornix, but the FynCA transgenic protein was also found accumulating in the cell bodies e.g., of large motor neurons in the brain stem and in nuclei such as the pontine nucleus. As tau is a substrate of Fyn we aimed to assess its phosphorylation in the FynCA mice. The phospho-Y18-tau-specific antibody 9G3 could not be employed because it is human tau-specific and does not detect murine tau phosphorylated at Y18 (Bhaskar et al., 2010). We therefore aimed to determine whether Ser/Thr-directed phosphorylation is increased in the presence of constitutively active Fyn. In human tau transgenic mice phosphorylation of Ser/Thr-epitopes of tau only occurs as the mice become older. We nonetheless analyzed 2 week-old FynCA mice using the phosphotau-specific antibody AT8 (Figure 5C), using non-transgenic littermates as controls (Figure 5D). We found that the mice were strongly phosphorylated at the AT8 epitope in fiber tracts as shown for the hippocampus, and pronounced AT8-immunoreactivity was also seen in cell bodies in several brain areas.

To determine whether expression of Δtau would ameliorate the FynCA pathology at least to a degree that would allow us to establish FynCA lines from the founder animals, we bred the FynCA founders with Δtau74 mice. Δtau is widely expressed in the brains of Δtau74-transgenic mice as revealed with the human tau-specific antibody HT7, whereas no expression is found in non-transgenic littermate controls (Ittner et al., 2010). Based on the co-transfection experiments in primary neurons (Figures 3G–K) and because of differences in expression patterns between Δtau in Δtau74 mice and FynCA in FynCA55 mice (which is in part due to different integration sites of the transgenes) (Figure 5), we expected only a subtle effect on survival. This is in fact what we observed because the rescue conferred by Δtau on the mortality of the FynCA mice was very subtle (Figure 4A).

### Increased phosphorylation of NR2b and tau in FynCa mice

We next obtained synaptosomal fractions adopting a previously established protocol (Ittner et al., 2010). Different from what we had done previously (Ittner et al., 2010), we used whole forebrains rather than pooled hippocampi. We sequentially extracted synaptosomes (*n* = 4 per genotype) from wild-type, FynCA and FynCA × Δtau (Tau^+/−^) mice and analyzed the PSD fraction containing the PSD for levels of Fyn, PSD-95, the NMDA receptor subunit 2b (NR2b), NR2b phosphorylated at Y1472, tau, and tau phosphorylated at the AT8 epitope S202/T205 and at the 12E8 epitope S262(/S356), using actin and PSD95 for normalization (Figures 6A,B). We also obtained total brain extracts, and as mentioned above, found that levels of transgenic FynCA expression were approximately six-fold higher than endogenous murine Fyn (Figure 6A).

When we analyzed the PSD fraction we detected massively increased levels of Fyn in FynCA compared with wild-type mice. When FynCA mice co-expressed Δtau, Fyn levels in the PSD fraction were not reduced, suggesting that either high levels of Fyn override the inhibition by Δtau in targeting Fyn to the spine (as observed for normal Fyn levels in Δtau74 mice) or non-overlapping expression patterns, or both. We found high levels of tau in the PSD fraction. Interestingly, whereas in total extracts obtained from FynCA mice, tau was massively phosphorylated at the AT8 epitope (tau phosphorylated at S202/ S205) (reflecting the histological data), we failed to detect tau phosphorylated at AT8 in the PSD fraction. This would suggest that if tau in the FynCA mice were phosphorylated at the AT8 epitope, levels in the spines would be very low. Interestingly, when FynCA mice were crossed onto a Δtau background, in total brain extracts, tau phosphorylation at the AT8 epitope was strongly reduced. Note: Δtau itself is not phosphorylated at the AT8 epitope, which is in line with previous observations showing that Δtau is not highly phosphorylated (Klein et al., 2002; Ittner et al., 2010). In agreement with these previous data, Δtau is not targeted to the PSD (Figure 6B). We also assessed tau phosphorylation at the 12E8 epitope (tau phosphorylated at S262 and possibly S356) and found that for the FynCA mice, 12E8 phosphorylation in the PSD fraction was increased compared with the fraction obtained from non-transgenic littermate controls. This increase was slight and not found in mice co-expressing FynCA and Δtau.

We further assessed NR2b phosphorylation and found that Y1472 phosphorylation was approximately four-fold increased in total extracts and in the PSD fraction obtained from FynCA compared with wild-type mice, and that levels were also increased when Δtau was co-expressed with FynCA.

## Discussion

Using two cellular systems we firstly showed that the palmitoylation signal is critical for the subcellular localization of the kinase Fyn: Mutating the two critical amino-terminal cysteine residues into serines abrogates localization of Fyn to the plasma membrane in HEK293 cells, and to dendritic spines in primary neuronal cultures. By mutating Y531 of Fyn to phenylalanine, we generated mice that express a constitutively active form of Fyn in neurons. These mice are characterized by early lethality around the age of weaning, a massively reduced weight and hyperactivity. We are also presenting preliminary evidence that constitutively active Fyn causes phosphorylation of endogenous murine tau at serine and threonine residues, as shown for the AT8 phospho-epitope of tau by both immunohistochemistry and Western blot analysis. Interestingly, tau in the spine seemed either not to be phosphorylated at AT8 at all or at least levels must be very low as we failed to detect AT8 phosphorylation in the “PSD blot” that was processed together with the “total blot.”

Fyn is a kinase that has been proposed to act as a signal integration hub that is involved in a vast number of cellular programs (Wang et al., 2013). In a pathological context such as AD, Fyn has been shown to integrate signals that ultimately lead to neuronal demise. Fyn mediates the downstream toxicity of Aβ by over-activating cellular receptors such as the NMDA receptor either directly or indirectly (Ittner et al., 2010). An additional role has been presented for the prion protein, PrP^c^. It has been shown that Aβ-induced dendritic spine loss requires both PrP^c^ and Fyn, and that in mice lacking PrP^c^, human familial AD transgene-induced convulsive seizures do not occur (Um et al., 2012). More recently, the metabotropic glutamate receptor 5 (mGluR5) was shown to function as a co-receptor of PrP^c^ (Um et al., 2013). Soluble Aβ was found to bind to PrP^c^ at dendritic spines where PrP^c^ forms a complex with Fyn, resulting in the activation of Fyn and subsequent Y18 phosphorylation, as shown with antibody anti-pY18 (Larson et al., 2012). However, how tau and Fyn are targeted to the dendritic spine is not understood. Two scenarios have been discussed previously (Larson et al., 2012): (i) Either Fyn is not bound to tau before entering the spine and activated Fyn then causes the phosphorylation of tau at Y18 resulting in its accumulation at the PSD. It is possible that at this stage phosphorylated Fyn might be bound to phosphorylated Tau binding to the SH3 domain of PSD-95, where it is ideally located to modulate NMDA receptor subunits such as NR2b at residue Y1472 (Ittner et al., 2010). (We found that FynCA is localized to the spine where it phosphorylates NR2b at Y1472.) (ii) The alternative scenario is that in the dendrite, Fyn is already bound to tau, which then translocates to the PSD to interact with PSD-95. There, another tyrosine kinase, Pyk2 (Köhler et al., 2013), could potentially activate Fyn and cause phosphorylation of tau at Y18. We found that increased levels of activated Fyn causes increased levels of phosphorylated Tau in the FynCA mice as shown for the AT8 epitope, although no increased phosphorylation was found for the spines. A time-resolved model for how Aβ via Fyn ultimately causes neuronal demise has been presented by Jannic Boehm, integrating the effects of Aβ on Fyn and STEP, a Fyn-phosphatase that eventually inactivates Fyn (Boehm, 2013).

Co-expressing Δtau and FynCA did not result in reduced levels of Fyn in the synaptosomal compartment. We had previously shown that transgenic expression of Δtau, which accumulates in the soma and is prevented from entering the dendrites, traps (endogenous) Fyn in the cell body and prevents it from entering the spines (Ittner et al., 2010). When FynCA is massively over-expressed, levels of Δtau seem not to be sufficiently high to prevent Fyn from entering the spines suggesting that manipulation of tau levels for therapeutic intervention may not be beneficial when Fyn is highly activated and/or its levels are increased, as may be the case under disease conditions. On the other hand it has been reported for AD brain by quantitative immunoblotting that Fyn levels were increased in the insoluble fraction and decreased in the soluble fraction. Soluble Fyn levels were directly correlated with cognitive scores and inversely correlated with tau-containing neurofibrillary tangle counts in the frontal cortex (Ho et al., 2005).

In spines it has been shown that hyperphosphorylated tau abnormally accumulates (Hoover et al., 2010). We have shown previously that in the presence of elevated levels of (phosphorylated) human tau, Aβ toxicity is exaggerated, most likely because, under these conditions, more Fyn enters the spine (Ittner et al., 2010; Ittner and Götz, 2011). In the presence of oligomeric Aβ tau not only changes its phosphorylation status but also alters its subcellular localization (Zempel et al., 2010, 2013). A role for tau phosphorylation specifically at the 12E8 epitope Ser262/Ser356 has been shown to be required for Aβ-mediated spine loss mediated via the CaMK2-AMPK signaling pathway (Mairet-Coello et al., 2013). We found in the present study that increased Fyn activity and levels in the spine do not necessarily result in increased phosphorylation, at least as shown for the AT8 epitope. This is different for the 12E8 phospho-epitope of tau, which reveals a slight increase in the FynCA PSD fraction compared to both non-transgenic littermate controls and mice co-expressing FynCA and Δtau. Further work needs to go into how Fyn regulates tau phosphorylation and what the requirements are for dendritic and spine localization of distinct phospho-pools of tau. Whether the results of our study would have been different if we had analyzed hippocampal fractions remains to be determined.

Earlier studies have been looking into transgenic mice that expressed FynB Y531F controlled by a CaMKII/SV40 expression vector (Kojima et al., 1998). The mice presented with higher seizure susceptibility. They also showed enhanced tyrosine phosphorylation of the NR2b subunit of the NMDA receptor. This was demonstrated by immuno-precipitating the NR2b subunit from the PSD fraction, running a blot, and probing it with a phospho-tyrosine-specific antibody. We confirmed this finding by probing the PSD fraction directly with an NR2b phospho-Y1472-specific antibody. Further work by Kojima and colleagues showed that an impairment of long-term potentiation in the absence of Fyn could be rescued by reintroducing FynB under control of the CamKII promoter (Kojima et al., 1997). When the constitutively active form of Fyn, Y528F was expressed in the epidermis using the keratin 14 promoter for expression (Li et al., 2007), 10% of homozygous mice died before weaning. Otherwise the mice were normal in size, and as they aged they developed spontaneous skin tumors. Together with our findings this reveals an important role for Fyn in peripheral organs and the brain.

Further work is required to determine how Fyn and tau are transported into the spine, which posttranslational modifications govern this process and which kinases are activated by the tyrosine kinase Fyn in order to cause serine/threonine-directed phosphorylation of substrates such as tau.

### Conflict of interest statement

The authors declare that the research was conducted in the absence of any commercial or financial relationships that could be construed as a potential conflict of interest.

## Supplementary material

Supplementary Movie **Movie (1 min) showing two FynCA transgenic mice (bottom) and two non-transgenic littermates (top) at 18 days of age**. The FynCA mice run in circles and keep up this hyperactivity for hours unless being put back to their litter.
